# Evidence for Countergradient Growth Adaptation and Jordan's Rule in a Southern Hemisphere Fish, the Atherinopsid *Odontesthes regia*

**DOI:** 10.1002/ece3.74084

**Published:** 2026-07-26

**Authors:** Hannes Baumann, Cristian Gallardo‐Escárate, Zofia A. Baumann, Miguel Araya, Cristian Azocar, Victor Aramayo, Marcelo E. Oliva, Mauricio A. Urbina

**Affiliations:** ^1^ Department of Marine Sciences University of Connecticut Groton Connecticut USA; ^2^ Interdisciplinary Center for Aquaculture Research (INCAR) Universidad de Concepción Concepción Chile; ^3^ Área Biología Marina y Acuicultura, Núcleo de Investigación Aplicada e Innovación en Ciencias Biológicas, Facultad de Recursos Naturales Renovables Universidad Arturo Prat Iquique Chile; ^4^ Doctorado en Conservación y Gestión de la Biodiversidad, Facultad de Ciencias Universidad Santo Tomás Santiago Chile; ^5^ Universidad Nacional Mayor de San Marcos Facultad de Ciencias Biológicas Lima Peru; ^6^ Dirección de Oceanografía y Cambio Climático Instituto del Mar del Perú Lima Peru; ^7^ Facultad Ciencias del Mar y Recursos Biológicos Universidad de Antofagasta Antofagasta Chile; ^8^ Departamento de Zoología, Facultad de Ciencias Naturales y Oceanográficas, Instituto Milenio de Oceanografía Universidad de Concepción Concepción Chile

**Keywords:** Chile, common garden experiment, growth capacity, latitude, Perú, temperature, vertebral number

## Abstract

We studied thermal growth reaction norms in larval/juvenile sea silversides ( *Odontesthes regia* ) from different populations along the Humboldt marine large ecosystem off the Chilean coast (20–41° S, ~2333 km), using a common garden approach. Two independent trials (years) consistently showed that offspring from a more northern, low latitude population (Iquique, 20° S) grew significantly slower than their conspecifics from a southern, higher latitude population (Dichato, 37° S) across all tested temperatures (14°C–23°C). This suggests that *O. regia* evolved countergradient growth variation, a form of local adaptation that works by accumulating faster‐growing genotypes in southern populations to counteract the negative phenotypic growth effects of higher‐latitude temperature conditions. Similar patterns have been found in Northern hemisphere fishes, particularly in silversides (Atherinopsidae), but to our knowledge, *O. regia* is the first documented case of countergradient growth adaptation in a Southern Hemisphere fish. The experiments also revealed consistent population differences in the length of hatchlings and early larval survival at low temperatures. In addition, we showed that fish from Peru and northern Chile (9–20° S) have approximately two fewer vertebrae than their conspecifics from south‐central Chile (24–41° S), indicating cogradient variation known as Jordan's Rule in fishes. The ubiquity of co‐ and countergradient variation worldwide suggests that these principles are broadly applicable to climate adaptation not only in space, but by inference also in time, thereby informing how organisms may evolve under global climate change.

## Graphical abstract

We performed common garden experiments on offspring of the sea silverside *Odontesthes regia* to characterize thermal growth reaction norms of different populations along the Chilean coast. This showed that the species exhibits local adaptation in the form of countergradient growth adaptation because higher‐latitude southern populations grew faster at all tested temperatures than their conspecifics from lower‐latitude northern populations. We also show that fish from northern populations have on average two fewer vertebrae than their southern conspecifics, which is a form of cogradient adaptation also known as Jordan's Rule in fishes.

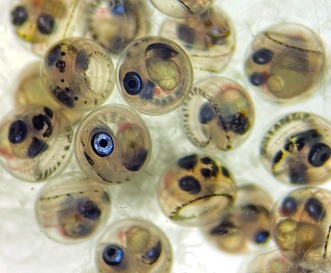

## 1 Introduction

How organisms adapt to environmental change is a fundamental and increasingly urgent question for science. Global climate change is certain to shift fitness landscapes for terrestrial and marine life, which is likely to respond with broad phenotypic and/or genotypic changes (Hoffmann and Sgrò 2011; Scheffers et al. 2016; Lotterhos et al. 2021). Evolutionary adaptation is occurring but predicting exactly how species and their traits will change in the future comprises one of the biggest scientific challenges of our time. One approach to the problem is to learn from spatial analogs, for example, from how organisms are already adapted to ubiquitous climate gradients across altitudes, depths, and latitudes. There, many species exhibit clines in morphological, physiological, and life history traits that arise from the interaction between phenotypic plasticity and genetic local adaptation (Conover and Schultz 1995; Sasaki and Dam 2019; Villeneuve et al. 2021).

**FIGURE 1:**
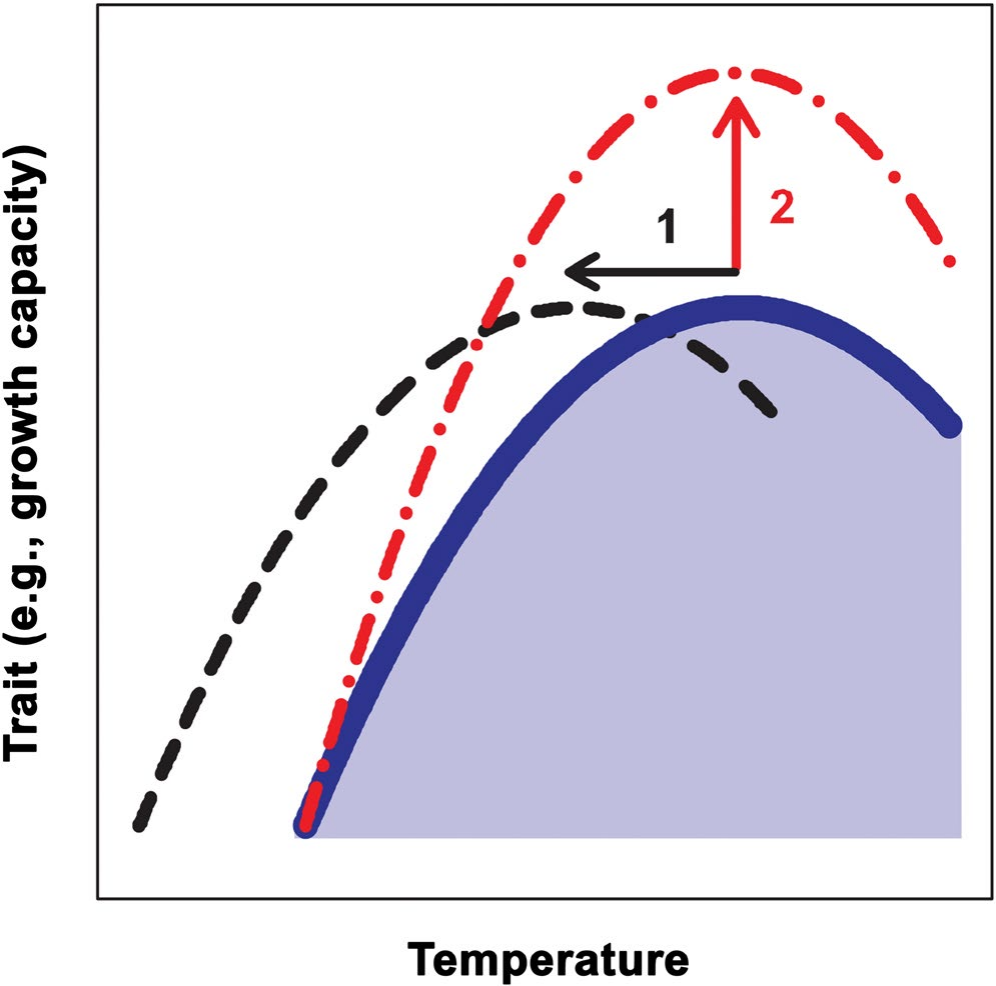
Consider two alternative ways local adaptation could modify the thermal reaction norm (filled shape) of a quantitative trait. Thermal adaptation (arrow 1) shifts thermal optima towards mean conditions at a location (*G × E*), whereas countergradient variation [arrow 2, Cov(*G*,*E*)] leads to genetic increases in trait expression over the entire thermal range without changes in thermal optima (modified after Baumann and Conover 2011).

Understanding adaptive trait variations requires distinguishing genetic from environmental influences; for example, by recognizing that a trait's phenotypic variance (*V*_P_) can be partitioned as *V*_P_ *= V*_G_ *+ V*_E_ *+ V*_G×E_ + 2Cov(*G*,*E*), where *V*_G_ is the variance component due to genotypic effects, and *V*_E_ is the variance due to environmental influences (Falconer and Mackay 1996). The latter is also known as the reaction norm, or phenotypic plasticity. A significant *V*_G×E_ interaction occurs when genotypes exhibit different, nonparallel trait reaction norms, and organisms therefore perform best in environments that they encounter most frequently (Figure 1; e.g., Lonsdale and Levinton 1985; Bronikowski 2000). The covariance term Cov(*G*,*E*), on the other hand, denotes that genotypes influencing a trait's phenotypic expression are nonrandomly distributed across a gradient of an environmental factor that also modifies the phenotype of this trait (Falconer and Mackay 1996; Conover et al. 2009). This term can be positive, when genetic and environmental effects reinforce each other, thereby leading to cogradient variation. Or it can be negative if genetic and environmental influences are in opposition to each other, leading to countergradient variation (Figure 1; see also Albecker et al. 2022). Cogradient variation results in highly noticeable phenotypic gradients across environmental clines (e.g., increase in vertebral number with latitude in fishes; Jordan 1891; McDowall 2008), but countergradient variation is often overlooked because it promotes phenotypic similarity across environmental gradients (Conover and Schultz 1995; Sparks et al. 2022).

Over past decades, examples for co‐ and countergradient adaptations have been found across most major taxa, but they are particularly well documented in marine and freshwater fishes (Conover et al. 2009). This is largely due to the extensive empirical work on a fish group known as new‐world silversides (Atherinopsidae), a family of approximately hundred marine and freshwater fishes endemic to North‐ and South‐America (Dyer and Gosztonyi 1999; Dyer 2006). Almost all are small‐bodied, short‐lived, ecologically important forage fishes—and several species have become valued laboratory models for eco‐evolutionary research (e.g., Silva et al. 2001; Brown et al. 2014; O'Leary et al. 2016). Experiments on Atlantic silversides ( *Menidia menidia* ) demonstrated that rearing temperature influences the sex ratio of offspring, the first case of temperature‐dependent sex determination in a fish (Conover 1984) that is also prevalent in South‐American freshwater silversides (Strüssmann et al. 1996; Yamamoto et al. 2014). Subsequent common‐garden experiments on *M. menidia* revealed that offspring from northern populations grow faster at all tested temperatures than their lower‐latitude conspecifics (32–44° N, ~1333 km) ‐ the first documented case of countergradient growth adaptation in a fish (Conover and Present 1990). Other common garden experiments have since confirmed and refined our understanding of co‐ and countergradient local adaptation in this and other northern hemisphere silverside fishes (Yamahira et al. 2006; Baumann and Conover 2011; Brown et al. 2012; Hice et al. 2012; Akopyan et al. 2026). However, to our knowledge, no comparable experiments have yet been conducted on silversides from the Southern Hemisphere; therefore, it is unknown whether they exhibit local adaptation across latitudinal gradients in form of co‐ and countergradient adaptation.

To fill this knowledge gap, we conducted thermal common garden experiments with the sea silverside *Odontesthes regia* (Humboldt 1821). We chose this species because it is exclusively marine and has a large latitudinal distribution from Peru to southern Chile (Dyer 2006), where mean sea surface temperature (SST) declines at a rate of 0.35°C latitude^−1^ (Baumann and Doherty 2013). Like most other silversides, *O. regia* are ecologically important forage fish (González and Oyarzún 2003; Herling et al. 2005) that are found year‐round in coastal waters, but during their reproductive season (September–November) adults occur in nearshore habitats to spawn (Plaza et al. 2011; Fierro et al. 2014). There, larvae and juveniles remain during summer and fall (Fierro et al. 2014), which limits their dispersal and therefore likely results in local, climate‐mediated selection conducive to local adaptation in fitness‐relevant traits. This is also supported by two recent studies detecting meristic and genomic differentiation between northern and southern *O. regia* populations (Deville et al. 2020, 2021).

The main goal of our study was to measure temperature‐specific length growth rates in *O. regia* offspring to characterize and compare thermal growth reaction norms of different populations (= latitudes). We therefore collected spawners from different locations across the Chilean coast and then reared their embryos to a juvenile size under a set of common temperatures. To increase statistical power, we conducted this common‐garden experiment twice, in two separate years. We hypothesized that *O. regia's* growth reaction norms would differ between populations, indicating local growth adaptation. Specifically, if *O. regia* exhibits local adaptation and it is comparable to Northern Hemisphere silversides, we would expect offspring from lower (northern) latitudes to grow more slowly at each temperature than their conspecifics from higher (southern) latitudes. In addition, we hypothesized that meristic traits also vary systematically along the same latitudinal temperature gradient. Specifically, we tested whether *O. regia* exhibits an increase in vertebral number with latitude, which is a form of cogradient adaptation consistent with Jordan's rule, a pattern widely documented in fishes.

## 2 Methods

### 2.1 Vertebral Number

**FIGURE 2:**
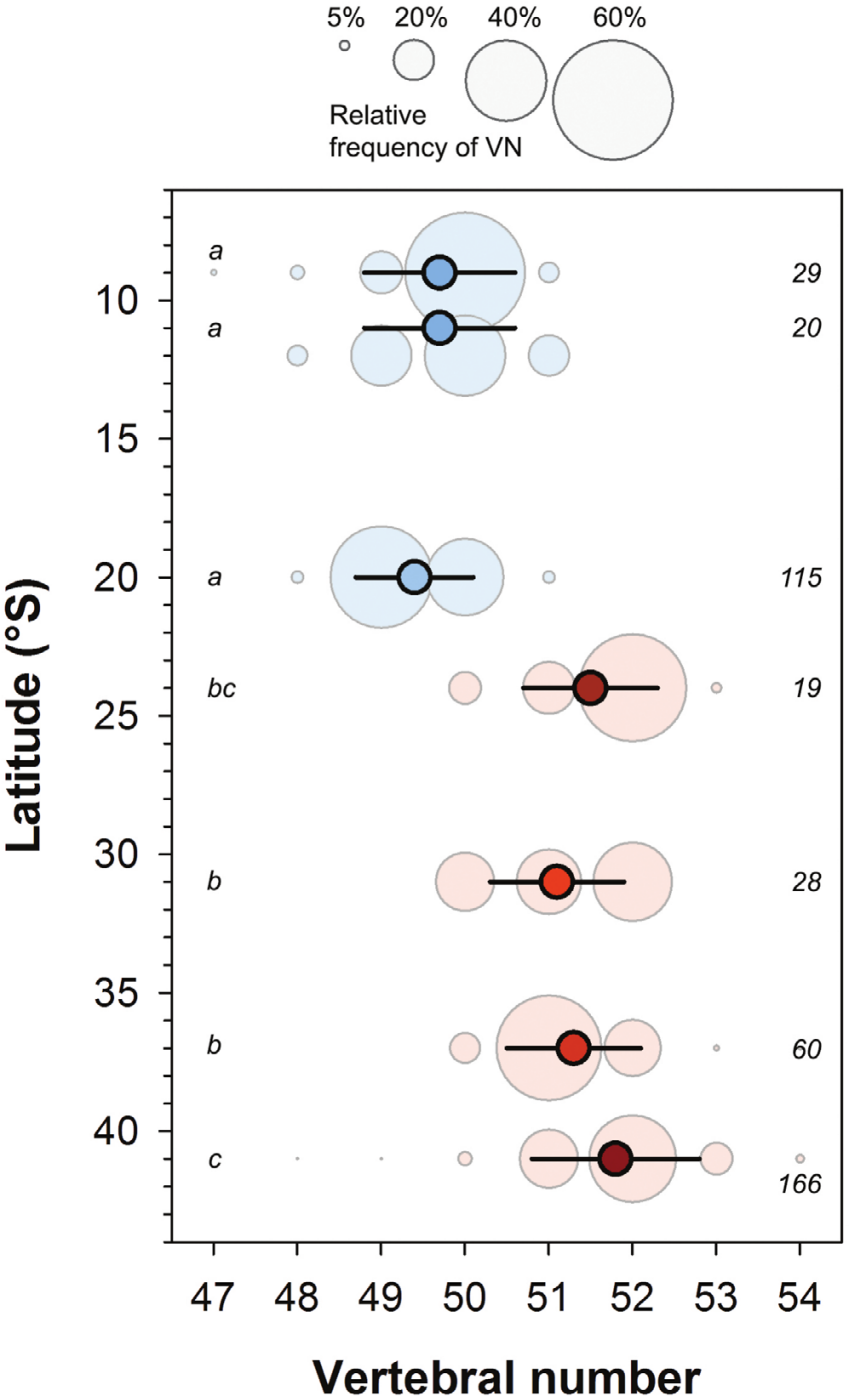
Number of vertebrae in *Odontesthes regia* adults collected in 2023 and 2025 from seven locations along the Peruvian and Chilean Pacific coast. Colored circles with horizontal error bars depict population‐specific means ± SD, while faint circles in the back are scaled to the relative frequencies of vertebral numbers for each population (each row adds up to 100%). Letters on the left denote significant differences between locations; numbers on the right denote analyzed (x‐rayed) specimens.

**FIGURE A1:**
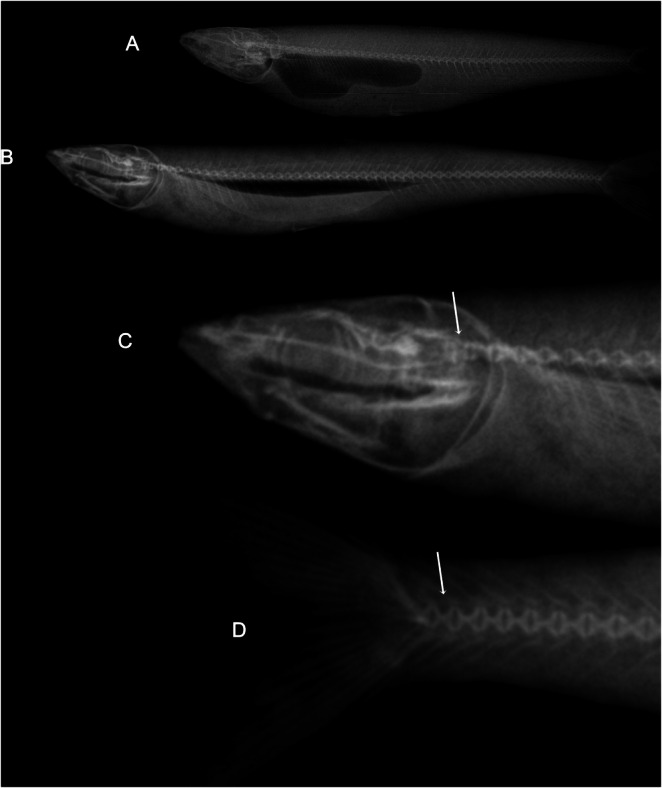
Examples of X‐ray images of adult *Odontesthes regia* used to count the number of vertebrae in each individual. (A) Specimen from Chimbote, Peru (9° S); (B) specimen from Dichato, Chile (37S), (C) enlargement of the head portion of B, with the arrow pointing to the first vertebra counted; (D) enlargement of the tail portion of B, with the arrow pointing to the last vertebra counted.

Adult *O. regia* were obtained from seven locations along the Pacific coast of Peru and Chile in 2023 and 2025 (9°–41° S, ~3555 km, Table 1). Specimens were caught with the help of local artisanal fishermen deploying gillnets (2–3 cm mesh) in nearshore waters during the night or early morning, then transported on ice and later stored frozen (−20°C) until further analysis. To determine the number of vertebrae, fish were imaged using digital radiography (x‐ray, Figure A1) at two different veterinarian clinics: the Centro Veterinario Diagnóstico (DiagnoPet) in Lima, Peru (Peruvian specimens) or the Oceanside Animal Hospital in Sandwich, MA, USA (Chilean specimens, using a VetRay SEDECAL, model A6504‐25). Exposure settings varied from 45kVp/4mAs and 50kVp/5mAs (Peruvian locations) to 60kVp/5.12mAs (Chilean locations). The digital x‐ray images were then analyzed with the open‐source software ImageJ (1.53a) using the multipoint tool to mark each vertebra between the basioccipital and the urostyle of each specimen (Figure A1). A total of 437 specimens were analyzed, but numbers varied between locations from *n* = 19 (24° S, Antofagasta) to *n* = 166 (41° S, Puerto Montt, Figure 2).

**TABLE 1:** Overview of *Odontesthes regia* sampling in 2023 and 2025, indicating populations used to measure vertebral number (VN) and/or to produce offspring for common garden experiments (CGE).

Population	Latitude (°S)	Sampling/fertilization date(s)		VN	CGE	Comments
		2023	2025			
Chimbote	9		11 Dec	✓		
Ancon	11		30 Oct	✓		
Iquique	20	15 Oct	5 Oct	✓✓	✓✓	
Antofagasta	24		12 Dec	✓		
Caleta Sierra	31	4 Oct		✓	✓	Offspring from single female in 2023; no spawners in 2025
Dichato	37	5 Oct	8 Oct	✓✓	✓✓	
Puerto Montt	41	26 Oct	5 Nov	✓✓	✓✓	Poor fertilization success (< 10%) in both years

### 2.2 Common Garden Experiments

**FIGURE A2:**
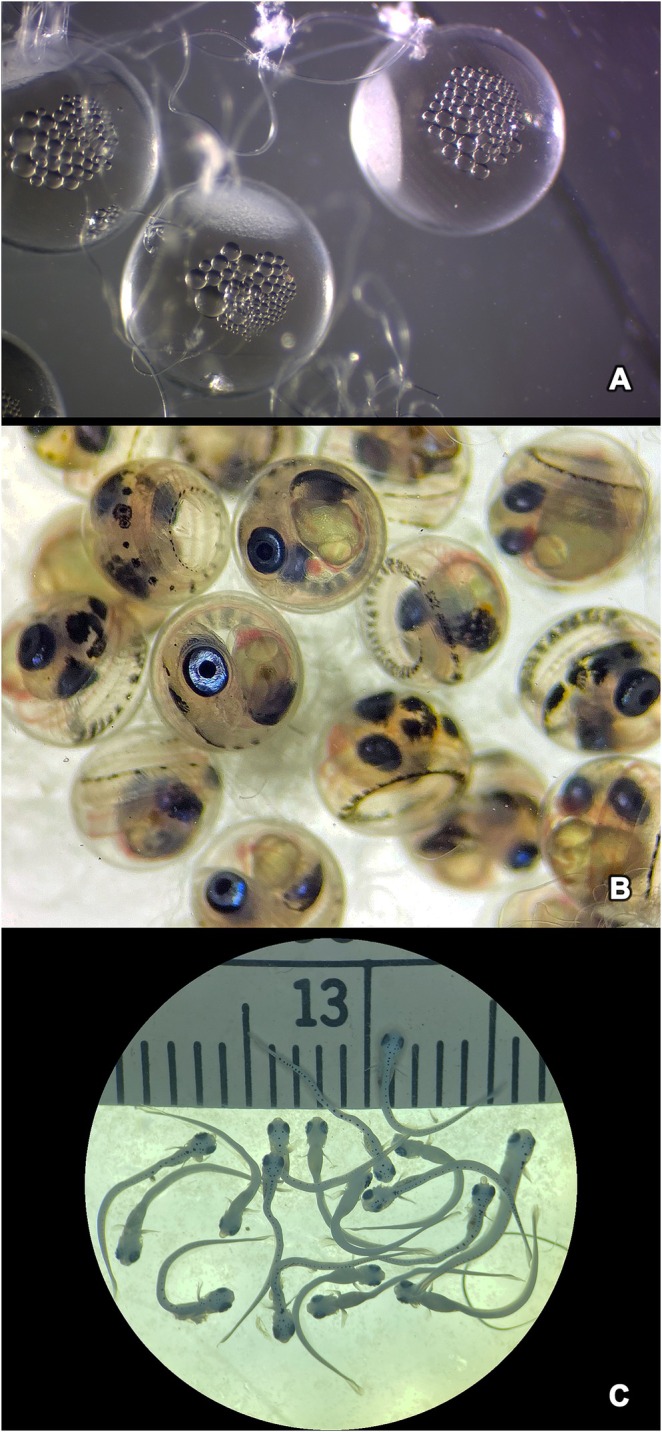
*Odontesthes regia* early life stages, (A) 24 h old embryos from the Iquique population, (B) embryos from the Dichato population in 2025 on the day of hatching at 14°C (22 days post fertilization), (C) newly hatched larvae of the Dichato population, imaged 24 h after preservation at 18× magnification for subsequent length measurements (1 mm scale).

**FIGURE A3:**
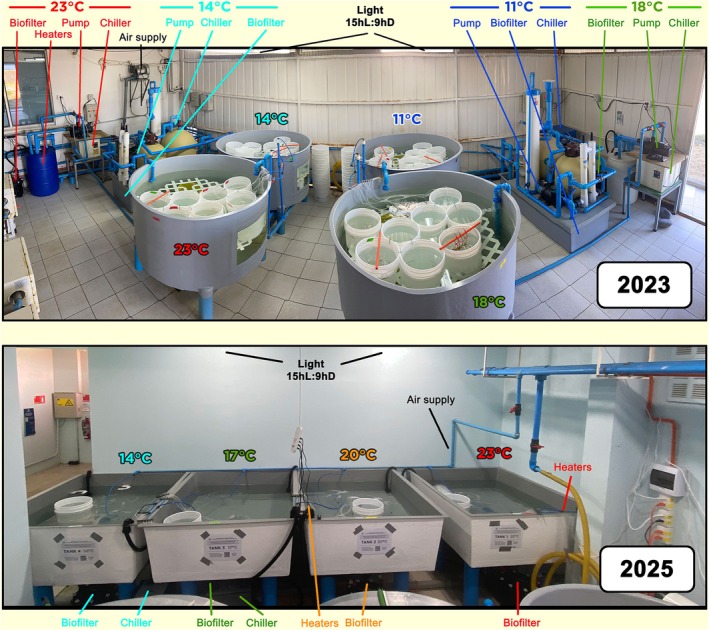
Labeled images of the common garden experimental setups at the Dichato Marine Research Station, housed in 2023 at the Interdisciplinary Center for Aquaculture Research (INCAR, picture taken on 7 October 2023) and in 2025 in the Experimental Marine Biology Laboratory (aka “Acuario”, picture taken on 4 October 2025) of the Universidad de Concepción.

To reveal potential genomic (i.e., latitudinal) differences in larval/juvenile thermal growth reaction norms, we conducted two independent common garden experiments in austral spring of 2023 and 2025. At each of four locations along the Chilean coast (Table 1), flowing‐ripe adults were obtained during the species' spawning season (September–November) as described above. Fish were separated by sex and kept alive for up to 1 h before being strip‐spawned into plastic dishes filled with clean seawater. At least 20 spawners per sex were used if available. Newly fertilized embryos (Figure A2a) were transported in cooler boxes to the rearing facility via car or airplane, and within 24 h post fertilization they were randomly distributed among temperature treatments. In both years, offspring were reared at the Dichato Marine Research Station of the Universidad de Concepción, where the experimental setup was housed at the Interdisciplinary Center for Aquaculture Research (INCAR) in 2023 and at the Experimental Marine Biology Laboratory (“Acuario”) in 2025 (Figure A3). In each experiment, we used four tanks of either 1200 L (2023) or 900 L each (2025) in recirculation mode to establish four controlled temperature treatments (2023: 11°C, 14°C, 18°C, 23°C; 2025: 14°C, 17°C, 20°C, 23°C) that were maintained via thermostats connected to in‐line chillers or aquarium heaters. Each tank was connected to a Fluval FX4‐250 canister filter or a bioball‐filled sump for biofiltration. Each tank held up to 12 rearing containers, allowing for three replicates per temperature for each of the four populations. The container design was identical to previous experimental work with silverside fishes (e.g., Baumann and Conover 2011; Murray et al. 2014); that is, we used round, white, 20 L polyethylene buckets equipped with individual airlines and mesh‐screened holes (150 μm) to guarantee oxygenation and water exchange with the surrounding tank while retaining fish and their food. Seawater of ~32 psu (29–34) was drawn from Coliumo Bay and filtered/UV‐sterilized before use in the experiment. Each tank was equipped with a HOBO Pendant MX2201 logger (Onset) that recorded temperatures every 30 min in 2023 and every 15 min in 2025. A photoperiod of 15 h light, 9 h dark was maintained throughout both experiments.

Starting at hatch and throughout the entire experiment, offspring were fed *ad libitum* rations of newly hatched brine shrimp nauplii ( *Artemia salina* , San Francisco strain, brineshrimpdirect.com), which we produced daily on‐site. *Ad libitum* or excess‐feeding conditions allow quantification of growth rates unaffected by food availability; hence, they are hereafter referred to as growth capacities (GC). *Ad libitum* conditions were maintained in each container by daily adding slightly more nauplii than the fish could consume over a 24 h period. After 1–2 days post‐hatch (dph), exactly 130 larvae were randomly distributed into three replicate rearing containers per population and temperature; this standardization avoided any density‐dependent growth effects and allowed quantification of survival from one experimental stage to the next. At the end of the first experimental stage, when larvae had reached on average ~15 mm total length (TL), 80 individuals were randomly chosen for further rearing, while the rest was sampled and preserved in 95% ethanol for TL measurements. At the second experimental stage (early juvenile stage of ~25 mm TL), 40 individuals were randomly chosen from each replicate for further rearing and the remainder preserved in 95% ethanol and later measured for TL. Rearing ended when juveniles had reached on average ~35 mm TL; then, all remaining survivors were sampled and preserved either individually (−20°C, 2023) or by replicate (95% ethanol, 2025) for final measurements. The ethanol was replaced once 24 h after initial fixation.

**FIGURE A4:**
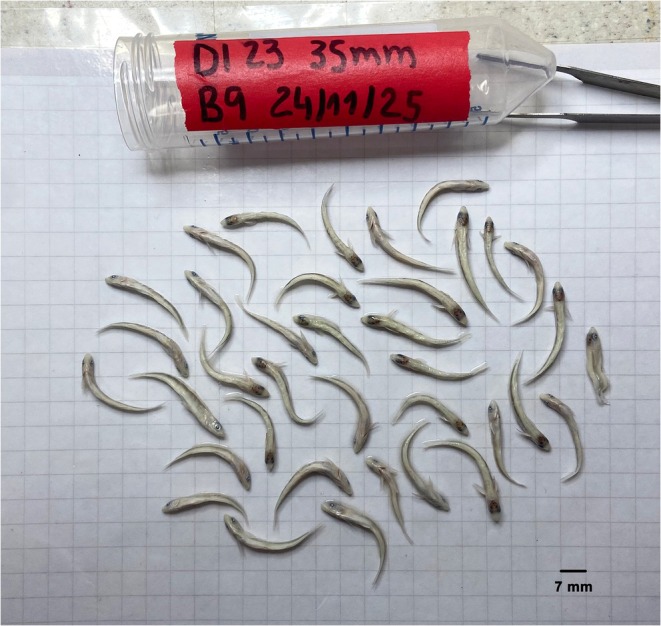
Example of an image used to digitally measure reared *Odontesthes regia* larvae and juveniles.

Adult spawners and non‐spawners were measured for TL using a ruler (nearest mm) and then preserved frozen (−20°C). Reared larvae and juveniles were sampled on their temperature‐ and population‐specific (1) day of peak hatch, (2) at ~15 mm, (3) at ~25 mm, and (4) ~35 mm TL. Hatch samples were photographed under a stereomicroscope at 18× magnification (Figure A2c); all other samples were photographed with an I‐Phone 11 on gridded (7 mm) background 24 h post preservation (Figure A4). The digital images were then analyzed with ImageJ 1.53a, using the segmented line tool to measure TL of all individuals (nearest 0.1 mm). Only in 2023, final samples (~35 mm TL) were measured for TL using calipers (nearest 0.1 mm) and blotted wet weight (nearest mg) and then preserved individually (−20°C) for further analyses to be reported elsewhere.

### 2.3 Data Gaps

**FIGURE A5:**
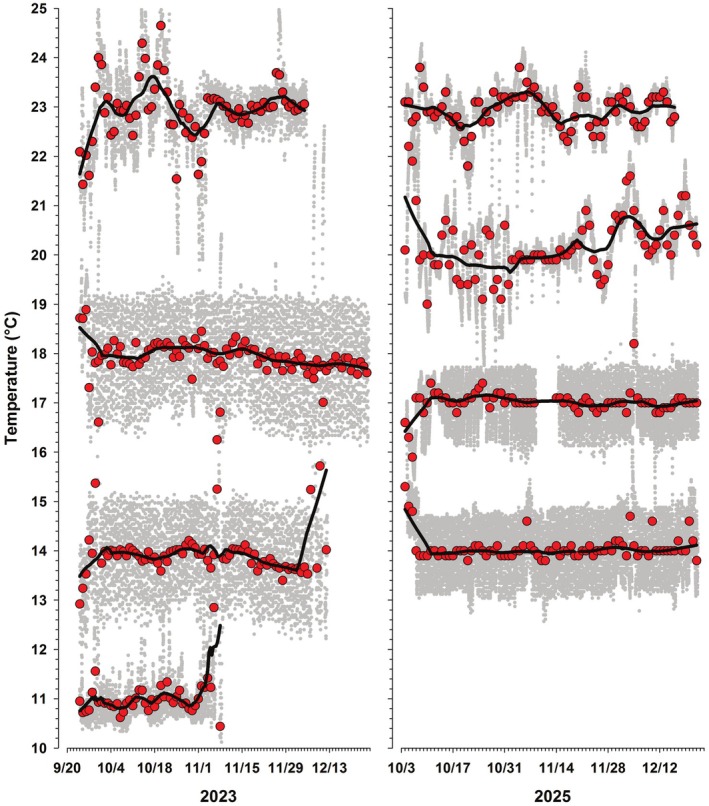
High‐resolution temperature data during the 2023 and 2025 common garden experiments at the Dichato Marine Research Station. Small gray circles show temperatures measured every 30 min (2023) or every 15 min (2025) via Onset HOBO loggers in each experimental tank. Larger red circles represent daily means, black lines are nonparametric LOESS interpolations (0.2 bandwidth). Chiller malfunction led to the premature termination of the 11°C and 14°C treatments in 2023.

Sampling of spawning ripe adults was largely unsuccessful in the mid‐latitudes of ~30° S (Coquimbo region). In 2023, all offspring from Caleta Sierra (CS, 31° S) originated from a single, flowing ripe female (strip‐spawned with five males), while in 2025 no spawning‐ripe fish were caught at all in this region. At the southernmost location of Puerto Montt (PM, 41° S), only a small fraction of adults was in truly flowing‐ripe condition on the sampling dates in 2023 and 2025, resulting in low fertilization success (< 10%), reduced stocking numbers, reduced replication, and potentially compromised growth patterns. For these reasons, we report CS and PM patterns separately (in the Appendix) from the empirically robust growth reaction norms obtained for the populations of Iquique (IQ, 20° S) and Dichato (DI, 37° S). In 2023, ambient laboratory temperatures exceeded the chillers' capacity to maintain 11°C on November 8th and 14°C on December 13th, forcing the early termination of these treatments (Figure A5). For logistical reasons, both experiments had to conclude by late December (20 Dec 2023; 22 Dec 2025), when fish in the coldest treatments had not yet reached 35 mm TL.

### 2.4 Data Analysis

After compiling all TL data, we first removed stunted or malformed fish via one‐sided outlier analysis. For that, we computed the mean (TL_mean_) and standard deviation of TL (TL_SD_) for each population (IQ, CS, DI, PM), temperature (11°C, 14°C, 17°C, 18°C, 20°C, 23°C), replicate (1, 2, 3), and sampling stage (hatch, 15, 25, 35 mm), then removed all specimens with a TL < TL_mean_—2 × TL_SD_ from further analyses. We then computed individual growth capacities (GC) i.e., TL growth rates (mm d^−1^) under *ad libitum* feeding conditions, since hatch (*GC1:* hatch‐15 mm, hatch‐25 mm, hatch‐35 mm), since the first subsample (*GC2:* 15–25 mm, 15–35 mm), and since the second subsample (*GC3:* 25–35 mm), using the population‐, temperature‐, and replicate‐specific means of the previous stage as the base for calculating the TL differential, divided by the days post‐hatch (*dph*) or since the first or second subsamples (*dp15, dp25*):

$${\text{GC1}}_{\text{ijkl}}=\left({\mathrm{TL}}_{\text{ijkl}}–\mathrm{TL}\_H_{\mathrm{kl}}\right)/{\mathrm{dph}}_{\mathrm{kl}}$$

$${\text{GC2}}_{\text{ijkl}}=\left({\mathrm{TL}}_{\text{ijkl}}–\mathrm{TL}\_15_{\mathrm{jkl}}\right)/\mathrm{dp}15_{\mathrm{kl}}$$

$${\text{GC3}}_{\text{ijkl}}=\left({\mathrm{TL}}_{\text{ijkl}}–\mathrm{TL}\_25_{\mathrm{jkl}}\right)/\mathrm{dp}25_{\mathrm{kl}}$$

for the *i*th individual of the *j*th replicate of the *k*th temperature of the *l*th population, where *TL_H* is the mean TL at hatch, *TL_15* is the mean TL at the 15 mm subsample, and *TL_25* is the mean TL at the 25 mm subsample. Because the unit of replication for all *GC* analyses was the replicate rearing container (not the individual fish), we subsequently averaged all *GCs* by stage, replicate, temperature, and population. To relate *GC*s to temperature, we first averaged raw temperature data by day, month, year and then used these averages to calculate actually experienced temperature means during each experimental stage (i.e., hatch‐15 mm, 15–25 mm, 25–35 mm) for each population and temperature‐treatment.

### 2.5 Statistical Analyses

Statistical analyses were conducted in SPSS Statistics (V20 IBM), using residual plots to verify parametric assumptions. All general linear models (GLM) included an intercept (error) term. Correlation analysis was used to relate vertebral number to latitude across all x‐rayed adult specimens (2023 and 2025), followed by a GLM that tested for between‐population differences in vertebral number with subsequent Bonferroni‐adjusted post hoc tests. Spawner TL was compared using a GLM with population (IQ, DI), sex (M, F), year (2023, 2025), and their interactive terms as fixed factors. Time to peak hatch (*H*, in days post fertilization, dpf) was modeled for IQ and DI as a 3‐parameter exponential decay function of rearing temperature *T* (*H* = y_0_ + a*e^−b^*^T^), followed by visual comparisons (within‐treatment replication only started post hatch). Analysis of larval length‐at‐hatch (*L*_H_) took the form of a GLM with population as a fixed factor, temperature (*T*) as covariate, year as a random factor, and a population*year interaction term. The population‐specific *T* versus *L*_H_ data were then fitted with a quadratic model of the form *L*_H_ = *a* + b*T + c*T ^2^. Replicate‐specific survival data (*S*, %) were first logit‐transformed [*S*_logit_ = log_10_(S*(1 − *S*)^−1^)], then used in an overall GLM with population, temperature, experimental period (hatch‐15 mm, 15–25 mm, 25–35 mm), and year as fixed factors (plus interactive terms), followed by separate GLMs for each period that included only population, temperature, and their interaction.

For *GC*, we used the mean for each replicate rearing container and statistically weighted it by the underlying number of measured individuals. Using at first only data from the 14°C and 23°C treatments that were assessed in both years, we tested for year effects on *GC1* with a GLM including population, temperature, experimental period, and year as fixed effects. We then used all data to construct six GLMs with only population, temperature, and their interaction as fixed factors: three models for *GC1* (hatch‐15 mm, hatch‐25 mm, hatch‐35 mm), two models for *GC2* (15–25 mm, 15–35 mm), and 1 model for *GC3* (25–35 mm).

## 3 Results

The vertebral number of *O. regia* adults ranged from 47 to 54 (*n* = 437, mean ± SD = 50.9 ± 1.3 vertebrae), and it was positively correlated with latitude (*R* ^2^ = 0.48, *p* < 0.001, Figure 2). Latitude had a significant effect on vertebral number (GLM; df = 6, *p* < 0.001), and post hoc tests showed that this was because specimens at the three northernmost locations (9–20° S) had approximately two fewer vertebrae (mean ± SD = 49.5 ± 0.8) than their conspecifics at the four southern populations (24–41° S, mean ± SD = 51.6 ± 1.0, Figure 2).

**FIGURE 3:**
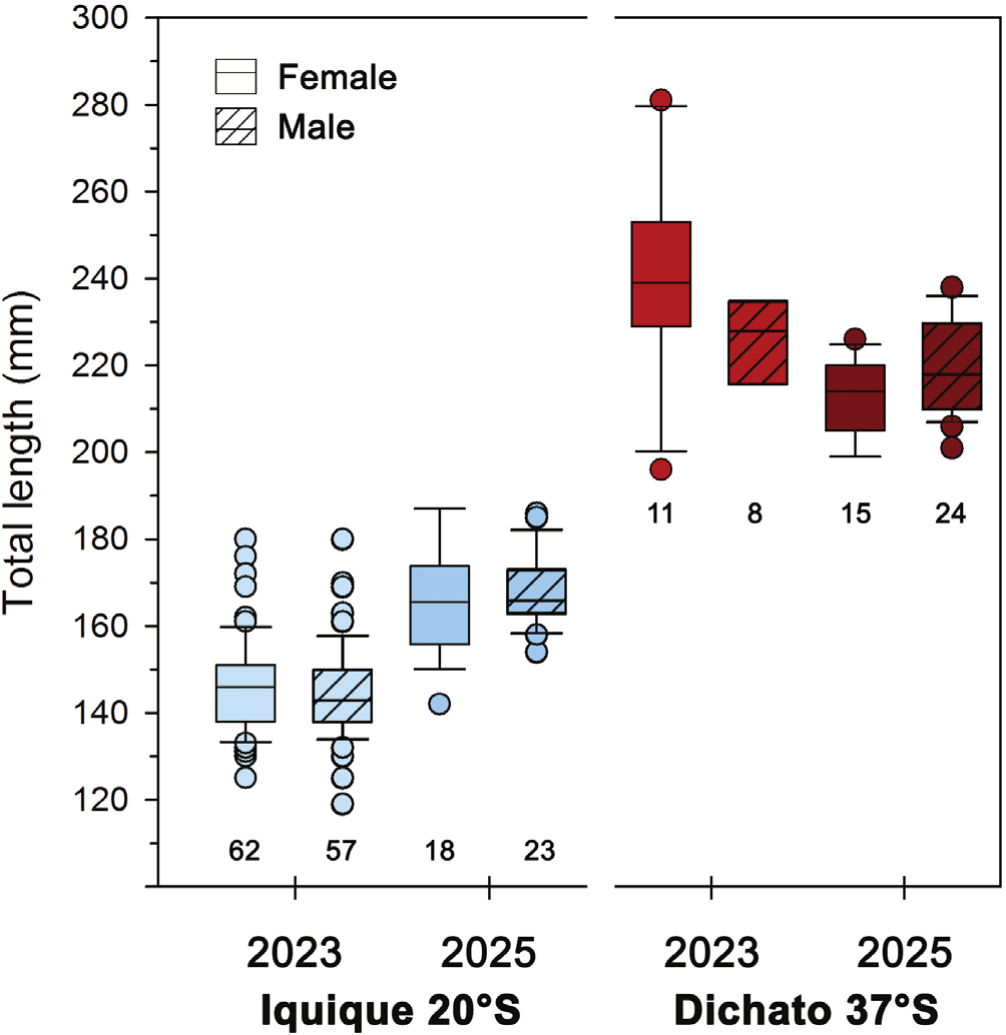
Box‐Wisker plots depicting TL distributions of *Odontesthes regia* females and males from Iquique (20° S, blue boxes/circles) and Dichato (37° S, red boxes/circles) used to produce offspring for common garden experiments in 2023 and 2025. Boxes denote 25th, 50th, and 75th percentiles, whiskers denote 10th and 90th percentiles, circles show outlier values. The number of measured adults is given below each box.

**FIGURE A6:**
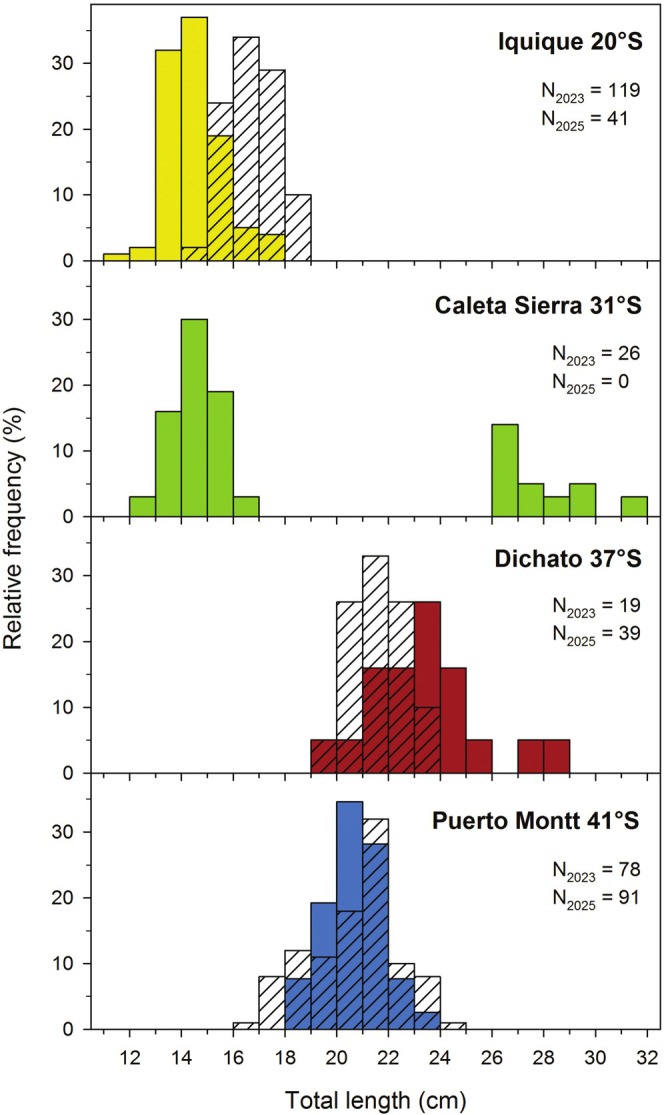
Relative TL frequency distributions of *Odontesthes regia* adults sampled from four populations along the Chilean Pacific Coast in 2023 (colored bars) and 2025 (hashed bars).

Spawners from IQ (20° S) and DI (37° S) that were used to fertilize embryos for the two common garden experiments differed significantly in TL (GLM, *p* < 0.001) irrespective of sex (*p* = 0.34) or year (*p* = 0.22), with IQ adults on average 68 mm smaller than DI adults (Figure 3). Within populations, males and females were of similar size (*P*_pop×sex_ = 0.19; TL_mean_ IQ = 156 mm, DI = 224 mm), but there was a population‐dependent year effect (*P*_pop×year_ < 0.001), because IQ spawners were smaller in 2023 (TL_mean_ = 145 mm) than in 2025 (TL_mean_ = 167 mm), while the DI spawners were larger in 2023 (TL_mean_ = 233 mm) than in 2025 (TL_mean_ = 216 mm, Figure 3). Adults from Puerto Montt (41° S) were of intermediate size with a mean TL of 211 mm, while those from Caleta Sierra (31° S) had a bimodal distribution containing both the smallest (TL_mean_ = 145 mm) and largest individuals caught overall (TL_mean_ = 278 mm, Figure A6).

**FIGURE 4:**
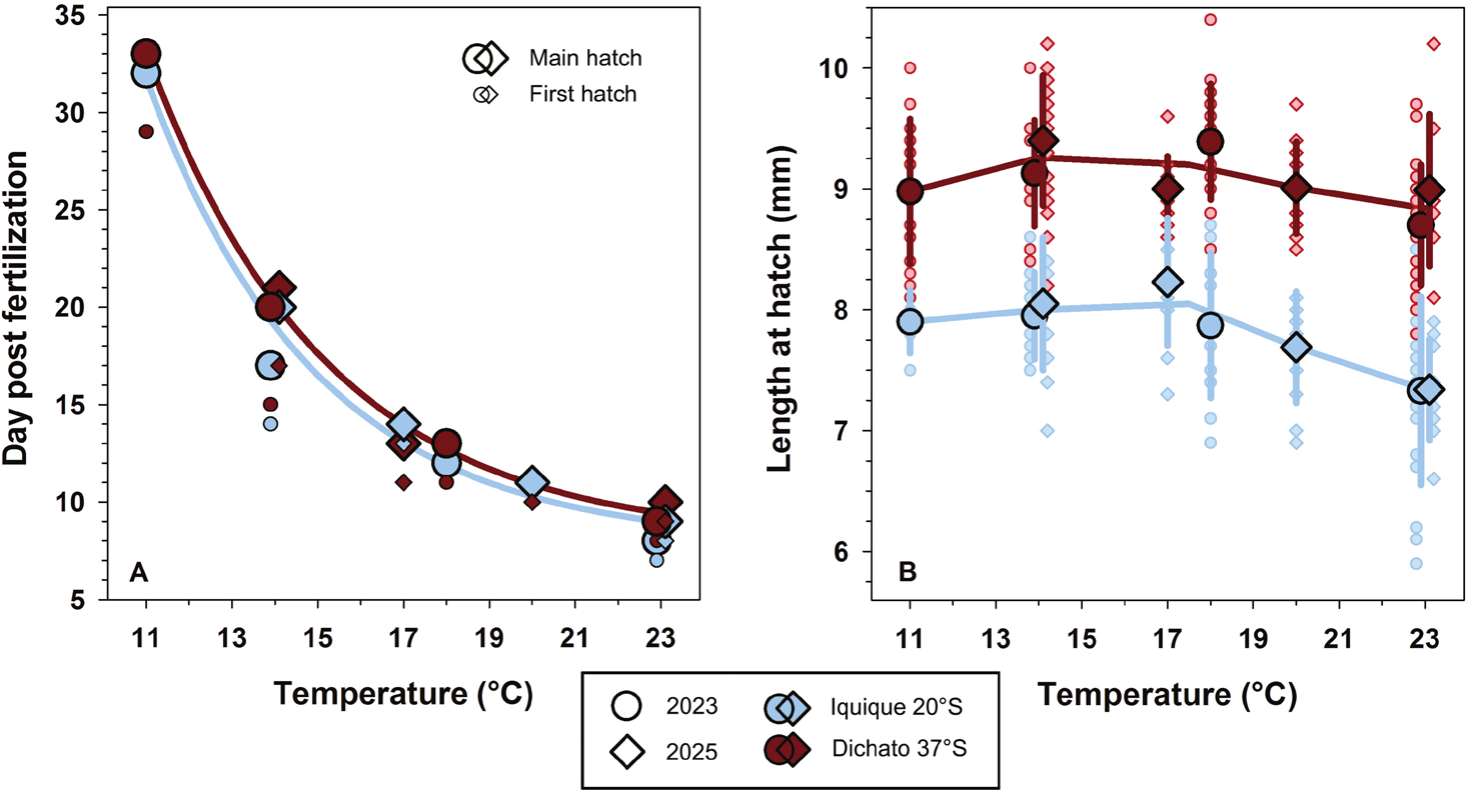
(A) Temperature‐dependent development time (fertilization to hatch, days) of *Odontesthes regia* embryos from Iquique (20° S, blue symbols) and Dichato (37° S, red symbols). Small and large symbols in depict the number of days to first and main hatch, respectively. Circles denote data from the 1st experiment in 2023, diamonds correspond to the 2nd experiment in 2025. Lines show exponential decay functions fitted to peak hatch (H, in dpf) versus temperature data across years ($R_{\mathrm{IQ}}^{2}$ = 0.983, $R_{\mathrm{DI}}^{2}$ = 0.995). (B) Temperature‐dependent length at hatch for *Odontesthes regia* larvae from Iquique and Dichato; small symbols show individual data points, big symbols show means ± SD for 2023 (circles) and 2025 (diamonds). Lines connect weighted means.

Under common garden conditions, embryos began hatching at 7–9 dpf and 29–30 dpf at the warmest (23°C) and coldest rearing temperatures (11°C), respectively, but peak hatch occurred at 8–9 dpf and 32–33 dpf, respectively (Figure 4A). Exponential decay functions for IQ and DI each explained > 98% of the variability (*p* < 0.001) and suggested, at most, a 1d overall difference between temperature‐dependent hatching in IQ and DI populations; however, this was not consistent across years or treatments (Figure 4A). Hatch length (*L*_H_) differed significantly between DI and IQ (GLM, *p* < 0.001, Figure 4B), because DI hatchlings (mean ± SD *L*_H_ = 9.1 ± 0.5 mm) were on average 1.3 mm (17%) larger than IQ hatchlings (mean ± SD *L*_H_ = 7.8 ± 0.6 mm). Year had a significant effect (*p* < 0.001), because hatchlings across temperatures were on average 0.17 mm larger in 2025 than in 2023. In both populations, *L*_H_ showed a significant (*p* < 0.001) dome‐shaped relationship with temperature (IQ: *L*_H_ = 5.5 + 0.34*T—0.01*T ^2^, *R* ^2^ = 0.23; DI: *L*_H_ = 6.6 + 0.34*T—0.01*T ^2^, *R* ^2^ = 0.14) with a predicted *L*_H_ maximum at ~16°C (Figure 4B).

**FIGURE 5:**
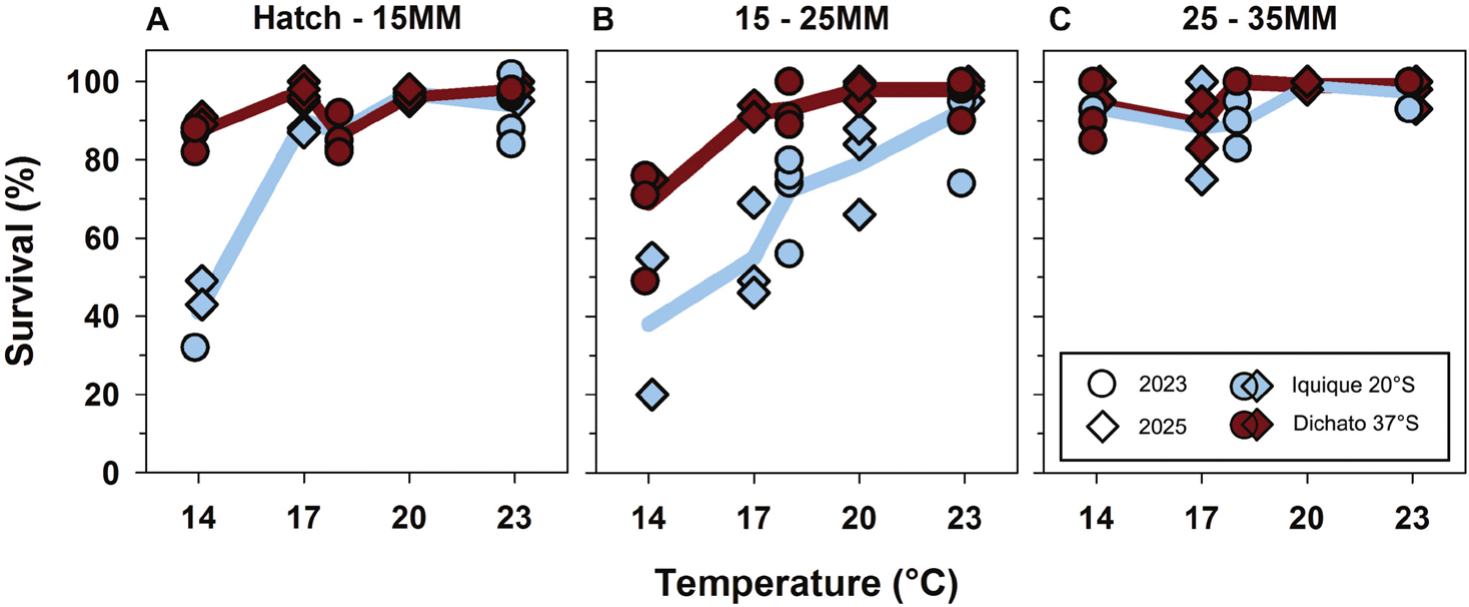
Temperature‐dependent survival (%) of *Odontesthes regia* larvae/juveniles from Iquique (20° S, blue symbols) and Dichato (37° S, red symbols) reared in common garden experiments in 2023 (circles) and 2025 (diamonds). Lines connect overall means across years. Survival was assessed during 3 periods; (A) from hatch to the 1st subsample at ~15 mm TL, (B) from the 1st to the 2nd subsample at ~25 mm TL, and (C) from the second subsample to the final sample at ~35 mm TL.

Survival was generally high throughout both experiments (range: 20%–100%, median = 95%; Figure 5). The overall GLM ($R_{\mathrm{adj}}^{2}$ = 0.43) showed no year effect on survival (*p* = 0.55), and there were no significant interactive terms. However, significant differences occurred between populations (*p* < 0.001), temperatures (*p* < 0.001), and experimental periods (*p* < 0.001). During the first period (hatch—15 mm), there was a weak population (*p* = 0.05) and a temperature effect (*p* = 0.004), because survival differed at the coldest temperature (14°C), with 82%–91% for DI larvae but only 32%–49% for IQ larvae (Figure 5A). From 15 to 25 mm, survival was strongly affected by population (*p* < 0.001) and temperature (*p* = 0.001), because both populations showed low and highly variable survival at the 14°C but increasingly higher, less variable survival at successively warmer temperatures. However, DI larvae always survived better than IQ larvae during this period (Figure 5B). During the final experimental period (25‐35 mm), survival was high (> 75%, mean = 95%) and statistically similar for IQ and DI regardless of temperature (Figure 5C).

**FIGURE 6:**
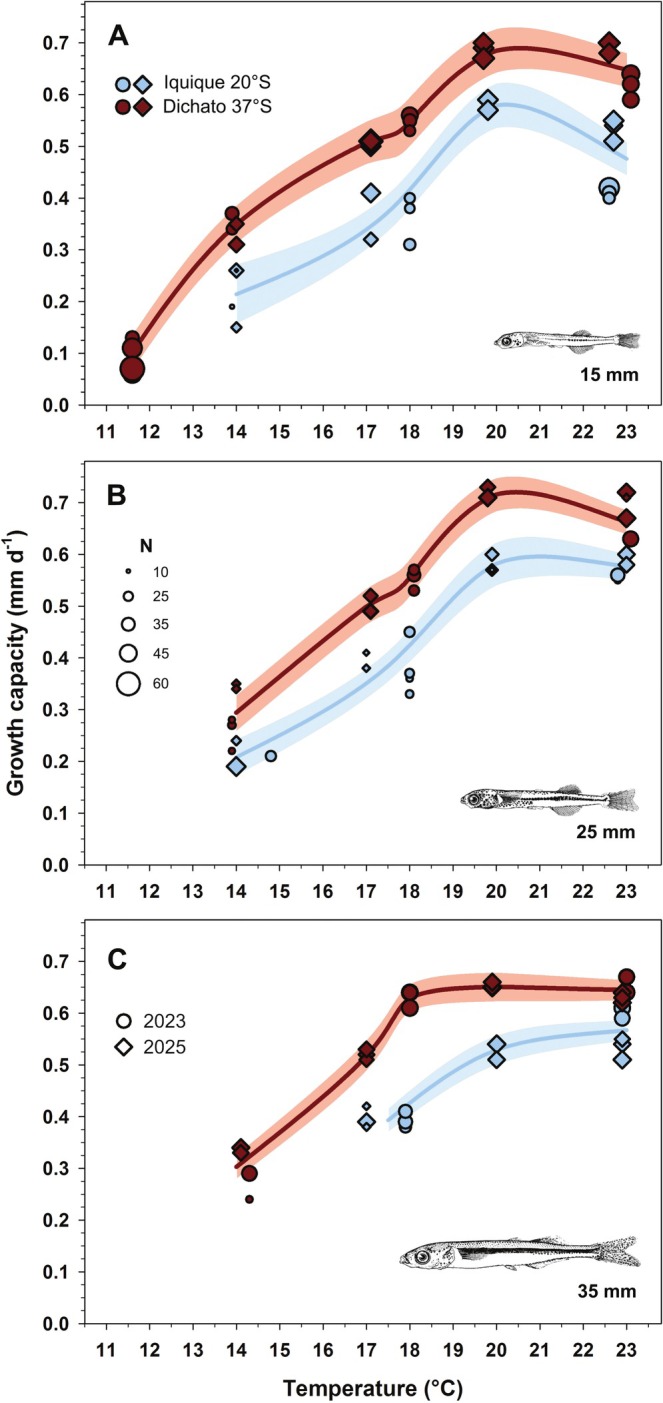
Temperature‐dependent growth capacities (GC1) of *Odontesthes regia* larvae/juveniles from Iquique (blue symbols) and Dichato (red symbols), reared in common garden experiments in 2023 (circles) and 2025 (diamonds) from fertilization to ~35 mm total length. Symbols depict replicate means scaled in size to the number of measured individuals. Lines connect weighted treatment means per population; shaded envelopes show 95% confidence intervals (for better visualization, a common mean/envelope is shown for the IQ_2025_ 17°C and IQ_2023_ 18°C data points). The three panels show GCs integrated from hatch to (A) the 1st subsample at ~15 mm TL, (B) the 2nd subsample at ~25 mm TL, and (C) the final sample at ~35 mm TL.

At the warmest (23°C) and coldest (14°C) temperatures assessed in both experiments, year had a significant effect on *GC1* (*p* < 0.001), because during more than half of all experimental periods IQ and DI offspring grew faster in 2025 than in 2023 (by 11%–35%, Table 2). When experiments were combined and all temperatures included, the 6 GLMs (3 × *GC1*, 2 × *GC2*, 1 × *GC3*) all showed strong population (*p* < 0.001) and temperature effects (*p* < 0.001), because at every temperature and experimental period *O. regia* offspring from Dichato (37° S) grew faster than their more northern conspecifics from Iquique (20° S, Figure 6). From hatch‐15 mm, for example, mean *GC1* in DI larvae increased from 0.09 mm d^−1^ at 11°C and 0.35 mm d^−1^ at 14°C to 0.69 mm day^−1^ at 20°C and 0.65 mm day^−1^ at 23°C, whereas *GC1* of IQ larvae only increased from 0.21 mm day^−1^ at 14°C to 0.58 mm day^−1^ at 20°C and 0.48 mm day^−1^ at 23°C (Figure 6A). The population effect was not temperature‐dependent (*P*_pop×temp_ = 0.06–0.35), because the two thermal growth reaction norms were approximately parallel to each other, and both suggested the *GC1* maximum to occur between 20°C–23°C (Figure 6A,B). Only during the last experimental period (25‐35 mm) did the *GC* difference between DI and IQ become less pronounced at 23°C, either due to an anomalous year effect (IQ_2023_ > IQ_2025_) or because IQ juveniles could indeed still grow slightly faster at 23°C than 20°C, but DI juveniles could not (Figure 6C).

**TABLE 2:** Mean growth capacities of *Odontesthes regia* offspring from Iquique and Dichato reared at 14°C and 23°C in common garden experiments in 2023 and 2025.

Population	Temperature °C	Stage	GC1 2023 mm day^−1^	GC1 2025 mm day^−1^
Iquique 20° S	14	H‐15	0.19	0.22
		H‐25	0.21	0.21
	23	H‐15	0.41	0.53*
		H‐25	0.56	0.59*
		H‐35	0.60*	0.54
Dichato 37° S	14	H‐15	0.36	0.34
		H‐25	0.26	0.35*
		H‐35	0.28	0.33*
	23	H‐15	0.62	0.69*
		H‐25	0.63	0.70*
		H‐35	0.66	0.63

*Note:* Growth was estimated from hatch (H) to a mean TL of 15 mm (H‐15), 25 mm (H‐25), and 35 mm (H‐35). Asterisks denote significant differences in GC1s between years (GLM, *p* > 0.05); bolded values indicate significantly higher GC1s (faster growth).

**FIGURE A7:**
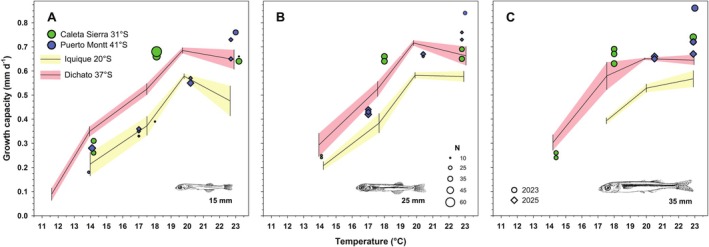
Temperature‐dependent growth capacities of *Odontesthes regia* larvae/juveniles from Caleta Sierra (green symbols) in 2023 and Puerto Montt (blue symbols) in 2023/2025 relative to the empirically robust thermal growth reaction norms determined for the Iquique (20S) and Dichato (37S) populations. Circles depict replicate means scaled in size to the number of measured individuals. Lines connect weighted treatment means per population; shaded envelopes depict 1 SD of the weighted mean. The three panels show growth capacities integrated from hatch to (A) to the 1st subsample at ~15 mm TL, (B) to the 2nd subsample at ~25 mm TL, and (C) to the final sample at ~35 mm TL. In both years, the fastest growth of larvae from Puerto Montt at the warmest temperature was consistent with the countergradient growth variation in this species; however, growth during the earlier growth periods and lower temperature treatments was more similar to Iquique.

**FIGURE A8:**
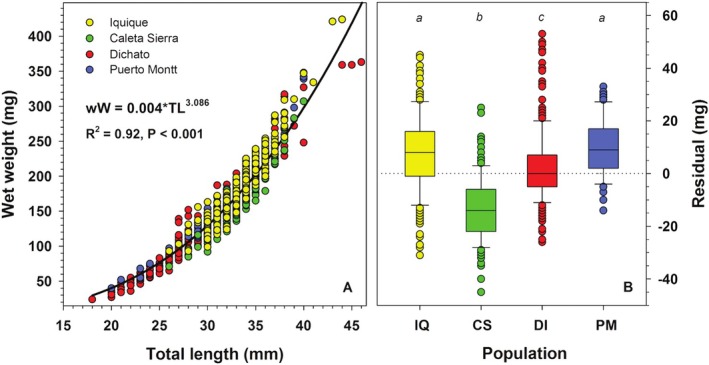
(A) Length‐weight relationship of laboratory‐reared *Odontesthes regia* juveniles from different populations along the Chilean coast (all temperature treatments combined). Each point represents an individual; the black line shows the exponential fit. (B) Residuals of the fitted exponential length‐weight relationship function, with boxes showing 25th, 50th and 75th percentiles, whiskers showing the 10th and 90th percentiles and symbols outside showing outliers. Letters denote significantly different populations (*p* < 0.05), based on a GLM with LSD post hoc tests.

*GC* data for the other two populations from CS (31° S) and PM (41° S) did not allow robust characterization of thermal growth reaction norms; however, we noticed that CS offspring grew similarly to DI offspring with consistently higher *GC1s* in 2023 than IQ offspring across all temperatures (Figures A7 and A8). In both years, PM offspring grew faster at 23°C than any other population (0.8–0.9 mm day^−1^), but at 14°C–20°C early larvae (hatch‐15 mm) showed surprisingly poor survival and grew similarly to IQ larvae (Figures A7 and A8).

## 4 Discussion

Our study used a common‐garden approach to quantify thermal growth reaction norms in *O. regia* larvae/juveniles, which we reared from different populations along the Chilean coast. The two independent years robustly demonstrated that offspring from a more northern, low‐latitude population (Iquique, 20° S) grew significantly slower than their conspecifics from a southern, higher‐latitude population (Dichato, 37° S) at all temperatures. Since phenotypic differences are genotypic differences under common garden conditions, this showed that *O. regia* exhibits local adaptation across its geographical distribution and underlying latitudinal temperature gradient. To reveal the specific type of adaptation, our experiments covered the species entire thermal range (11°C–23°C), thereby intentionally exceeding what individual populations experience in south‐central (11°C–16°C) or northern regions of Chile (18°C–23°C; Baumann and Doherty 2013; Deville et al. 2020). One might intuitively expect that this led to each population performing best within, but poorer outside, its natural thermal range, a pattern known as the “adaptation‐to‐temperature model” (Yamahira and Conover 2002). But that did not happen here, because offspring from all populations grew fastest at the same, warm temperature, and their thermal growth reaction norms did not cross. Such a pattern suggests a type of local adaptation known as countergradient variation, where genetic and environmental influences on a trait oppose each other (Conover and Schultz 1995). In *O. regia* , it achieves adaptation by accumulating faster growing genotypes in southern populations, thus counteracting the phenotypic, negative growth effect of lower temperatures at higher latitudes. Why this type evolved over others remains speculative; perhaps countergradient variation requires the least amount of molecular reorganization and can therefore arise first between conspecifics (Baumann and Conover 2011). By now, examples have been found in many taxa, including in plants (e.g., Chapin and Chapin 1981), mollusks (e.g., Villeneuve et al. 2021), crustaceans (e.g., Campos et al. 2009), insects (e.g., Blanckenhorn 1991), fish (e.g., Blanchard et al. 2024), amphibians (e.g., Berven 1982), and reptilians (e.g., Smith et al. 1994). The principle could be even more common; it is easily overlooked because it obscures rather than promotes phenotypic differences across environmental gradients (Conover et al. 2009). Gardiner et al. (2010) detected it in the contrasting respiration rates of cardinal‐ and damselfishes in northern versus central Great Barrier Reef sites. However, to our knowledge, the atherinopsid *Odontesthes regia* is the first documented case of countergradient growth adaptation in a southern hemisphere fish.

More conclusive proof of this evolutionary principle in *O. regia* would require more than two populations, which is why we intended to test both a more southern location at 41° S and one at an intermediate latitude of ~30° S. In the first case, our collections likely occurred too early in October/November (Table 1) when spawners there were mostly still unripe, unlike those at 37° S. Logistical constraints precluded reattempts in our study, but future work will address these challenges. We did obtain some, albeit inconclusive data for this location: on one hand offspring grew fastest at 23°C (a temperature they do not naturally experience at 41° S) just like all other populations, and they were the fastest‐growing offspring overall at 23°C—exactly as countergradient variation predicts. But the poor survival and lower growth at colder temperatures were not consistent with any form of adaptation; at this point, they remain an unexplained experimental artifact.

In contrast, the challenge at Chile's midlatitudes ~30° S (Coquimbo) was to even find *O. regia* adults at all in either experimental year. An earlier expedition in October 2022 readily encountered fishermen catching running ripe adults there, but during the austral spring of 2023 the fish were conspicuously absent from nearshore habitats. This was likely due to coast‐wide shifts in assemblages in response to anomalously high SSTs caused by the strong 2023 El Niño, which also brought severe drought and wildfires to central Chile (Cordero et al. 2024; Martinez‐Villalobos et al. 2024). We nevertheless obtained some offspring that year from one very large female crossed with only five males, all haphazardly collected from a small cove south of Coquimbo (Caleta Sierra, 31° S). While not representative of a larger population, these growth data are partially consistent with countergradient variation, because offspring showed higher growth capacities at all temperatures than their lower‐latitude conspecifics from 20° S (Iquique), but they did not grow more slowly than fish further south from 37° S (Dichato).

Two years later (2025), spawning *O. regia* had still not returned to the nearshore waters of central Chile (pers. observation, Valparaiso to Coquimbo), although the El Niño Southern Oscillation (ENSO) index became weakly negative/neutral again in 2025.^1^ Our interpretation is that *O. regia* 's geographical distribution might in fact be discontinuous in many, or even most years, with southern and northern population clusters largely separated by nonoccurrence at central Chilean latitudes. Other studies have already recognized the region around 30° S as a major genetic break for crustacean, mollusk, and echinoderm species (Haye et al. 2014; McKeown et al. 2019), and recent molecular evidence specifically for *O. regia* further corroborates this idea. Mitochondrial and nuclear DNA markers in *O. regia* both suggested small but temporally consistent genomic differentiation between populations from south‐central Chile (37–42° S) vs. northern Chile and Peru (6–20° S), implying limited gene flow across the hypothesized genetic break at 30° S (Deville et al. 2021). Our experiments provided further evidence of genetic differences in early life history traits between the two regions. Apart from larval growth capacities, we also found larger hatch lengths implying faster embryonic growth at higher latitudes, which is consistent with spatial patterns reported for anchoveta *Engraulis ringens* (Llanos‐Rivera and Castro 2004, 2006). Larval survival also showed signs of adaptive differentiation between northern and southern *O. regia* populations, as the former survived less well at low temperatures (14°C) than the latter despite the fact that hatchling size remained similar across temperatures. High temperatures (23°C), however, resulted in uniformly high survival in all populations.

Other differences between northern and southern *O. regia* populations, for example, in adult body size and meristic traits, were detectable without experimentation, because local adaptation in these traits often takes the form of co‐gradient variation (Conover et al. 2009). It occurs when genetic and environmental influences reinforce each other, thereby producing highly noticeable phenotypic gradients that have long been known as ecogeographical rules. For example, the observed increase in body size with latitude in *O. regia* (Deville et al. 2020; this study) is consistent with *Bergmann's Rule* (Bergmann 1847; Belk and Houston 2002), while Deville et al. (2020) suggested that a latitudinal increase in the number of gill rakers in *O. regia* conforms to *Jordan's Rule*. Technically, that rule only refers to latitudinal changes specifically in vertebral number (Jordan 1891; McDowall 2008), which had not been shown yet prior to this study. Consistent with the taxonomic literature we counted 47–54 vertebrae overall in *O. regia* (Dyer and Gosztonyi 1999), and we confirmed *Jordan's Rule* in *O. regia* by demonstrating that southern populations have, on average, 2 more vertebrae than northern populations. Interestingly, the fish collected at 24° S (Antofagasta) grouped with those from south‐central populations (31–41° S) – implying that the genetic break either occurs further north (between 20 and 24° S) than previously hypothesized (Deville et al. 2021) or that genetic transitions are different for different traits as recently discovered in a northern hemisphere silverside species (Akopyan et al. 2026).

Overall, our work showed that *O. regia* is an excellent fish model for many types of laboratory studies (e.g., Silva et al. 2001), not only because adults can be sampled and strip‐spawned with relative ease, but also because rearing offspring is straightforward and feasible with basic husbandry techniques. For example, because *O. regia* eggs/embryos are about twice as large (~2 mm) than those of most marine fish (Barneche et al. 2018), the larvae also hatch at comparatively large sizes (7–9 mm) and can be fed immediately with *Artemia* nauplii (Chirinos de Vildoso and Chumán 1964). This makes *O. regia* similarly useful to eco‐evolutionary studies as other species of new world silversides (family Atherinopsidae), including those in South‐American freshwater systems (*O. argentinensis, O. hatcheri*: Strüssmann et al. 1996; *O. bonariensis* : Chalde et al. 2011) or those along North Americas Atlantic and Pacific coasts ( *Atherinops affinis* : Baumann and Conover 2011; *M. menidia* : Murray et al. 2014; *Leuresthes tenuis* : Siegfried and Johnson 2023).

With respect to local adaptation, the Atlantic silverside *M. menidia* stands out as one of the best studied fish models in the ocean (Conover 2025). Decades of empirical studies have demonstrated not merely the existence of co‐ and countergradient variation in a number of traits but also their adaptive significance, trade‐offs, and underlying genomic mechanisms (Hice et al. 2012; Akopyan et al. 2022) – which likely apply at least partially to *O. regia* as well. For example, the generally inverse relationship between growth and mortality in the early life stages of fish makes selection for faster growth at higher latitudes an intuitive finding. But if so, the persistence of slower‐growing genotypes at lower latitudes would be puzzling, unless there are trade‐offs to fast growth. Work on *M. menidia* has revealed such trade‐offs, showing that slower‐growing genotypes have superior predator avoidance, because their greater metabolic scope allows for higher critical swimming speeds compared to fast growers (Munch and Conover 2003; Munch et al. 2003). Similar adaptive trade‐offs likely account for countergradient growth adaptation in *O. regia* ; however, there are also important differences. In *M. menidia* , the thermal growth reaction norms show a pronounced latitude × temperature interaction, but in *O. regia* they did not. In the former, higher latitude populations grow disproportionally faster than lower latitude conspecifics at warmer temperatures, likely to compensate not just for the latitudinal temperature effect on growth but even more so for a decrease in the length of the growing season (Schultz et al. 1998). This arises, because the North‐American Atlantic coast has the worlds steepest latitudinal climate gradient (0.91°C lat^−1^) with large changes between average winter lows and summer highs (Baumann and Doherty 2013). Conversely, average SSTs decline much more gradually along the South American Pacific coast (0.35°C lat^−1^), with minimal latitudinal variation in seasonal patterns, and there is no evidence that the growing season is truncated in *O. regia*. We conclude that both silverside species show the same general type of local growth adaptation, but that the specific patterns likely reflect the strength and nature of underlying latitudinal climate gradients (Baumann and Conover 2011).

Our conclusion is similar for co‐gradient adaptation, here shown for vertebral number, because again the degree of change ( *M. menidia* : +7 vertebrae from 32 to 46° N; *O. regia* : +2 vertebrae from 9 to 41° S) – appears to resemble the strength of the underlying latitudinal climate gradient (Baumann et al. 2012). The adaptive significance of *Jordan's Rule* continues to be explored; however, work on sticklebacks has suggested that more vertebrae confer better swimming abilities in colder, more viscous waters at higher latitudes (Swain 1992a, 1992b; Reimchen and Cox 2016).

In conclusion, our study suggested that co‐gradient and countergradient variation comprise ubiquitous intraspecific forms of local adaptation in fishes across spatial climate gradients. Understanding how such patterns evolve and what trade‐offs are involved has undoubtedly become of great urgency to mankind, given that climate change now also occurs across temporal scales and at an accelerating rate. Recent studies have demonstrated that the same principles also apply to evolutionary adaptation in time (Garant et al. 2004; Wilson et al. 2007); suggesting that “space‐for‐time” analogies are one important tool for scientists to anticipate how organisms will adapt to anthropogenic climate change. Spatial analogs promise to elucidate how evolution will ultimately respond to climate change, what selective factors are most important, and what physiological mechanisms are underlying adaptation, even if they cannot reveal evolutionary rates.

## Author Contributions

**Hannes Baumann:** conceptualization (equal), data curation (equal), formal analysis (equal), funding acquisition (equal), investigation (equal), methodology (equal), project administration (equal), supervision (equal), validation (equal), visualization (equal), writing – original draft (equal), writing – review and editing (equal). **Cristian Gallardo‐Escárate:** conceptualization (supporting), investigation (supporting), project administration (supporting), resources (supporting), supervision (supporting), writing – review and editing (supporting). **Zofia A. Baumann:** data curation (supporting), funding acquisition (supporting), investigation (supporting), project administration (supporting), resources (supporting), writing – review and editing (supporting). **Miguel Araya:** investigation (supporting), methodology (supporting), resources (supporting), supervision (supporting), writing – review and editing (supporting). **Cristian Azocar:** investigation (supporting), methodology (supporting), resources (supporting), writing – review and editing (supporting). **Victor Aramayo:** data curation (supporting), resources (supporting), writing – review and editing (equal). **Marcelo E. Oliva:** investigation (supporting), resources (supporting), writing – review and editing (supporting). **Mauricio A. Urbina:** conceptualization (supporting), funding acquisition (equal), investigation (supporting), project administration (equal), resources (supporting), supervision (supporting), writing – review and editing (supporting).

## Funding

This work was funded by NSF‐OCE grant #2313288 to H.B., Z.B., and M.U.F. and a seed grant from the Universidad de Concepción to H.B. and M.U.F.

## Ethics Statement

All experimental procedures were jointly reviewed and approved by responsible committees at the Universidad de Concepción (Comité de Ética, Bioética y Bioseguridad) and at the University of Connecticut (Institutional Animal Care and Use Committee) under the protocol #CEBB 1501–2023.

## Conflicts of Interest

The authors declare no conflicts of interest.

## Data Availability

The source data for this study are accessible from the repository of the Biological and Chemical Oceanography Data Management Office (BCO‐DMO) at https://www.bco‐dmo.org/dataset/956677 and are citable as: Baumann and Baumann (2026).

## Endnotes

^1^

https://iri.columbia.edu/our‐expertise/climate/enso/.
